# Endovascular and Surgical Treatment of Unruptured MCA Aneurysms: Meta-Analysis and Review of the Literature

**DOI:** 10.1155/2014/348147

**Published:** 2014-04-01

**Authors:** Spiros L. Blackburn, Abdelrahman M. Abdelazim, Andrew B. Cutler, Kevin T. Brookins, Kyle M. Fargen, Brian L. Hoh, Yasha Kadkhodayan

**Affiliations:** ^1^University of Florida, Department of Neurosurgery, P.O. Box 100265, Gainesville, FL 32610-0265, USA; ^2^Assiut University, Department of Radiology, Assiut 71515, Egypt; ^3^University of Florida, College of Medicine, Gainesville, FL 32603, USA; ^4^Division of Interventional Neuroradiology, Abbott Northwestern Hospital, Minneapolis, MN 55407, USA

## Abstract

*Introduction*. The best treatment for unruptured middle cerebral artery (MCA) aneurysms is unclear. We perform a meta-analysis of recent publications to evaluate the results of unruptured MCA aneurysms treated with surgical clipping and endovascular coiling. *Methods*. A PubMed search for articles published between January 2004 and November 2013 was performed. The R statistical software package was used to create a random effects model for each desired incidence rate. Cochran's *Q* test was used to evaluate possible heterogeneity among the rates observed in each study. * Results*. A total of 1891 unruptured MCA aneurysms, 1052 clipped and 839 coiled, were included for analysis. The complete occlusion rate at 6–9 months mean follow-up was 95.5% in the clipped group and 67.8% in the coiled group (*P* < 0.05). The periprocedural thromboembolism rate in the clipping group was 1.8% compared with 10.7% in the aneurysms treated by coiling (*P* < 0.05). The recanalization rate was 0% for clipping and 14.3% for coiling (*P* = 0.05). Modified Rankin scores of 0–2 were obtained in 98.9% of clipped patients compared to 95.5% of coiled (NS). *Conclusions*. This review weakly supports clipping as the preferred treatment of unruptured MCA aneurysms. Clinical outcomes did not differ significantly between the two groups.

## 1. Introduction

Endovascular coiling has emerged as an option in the management of intracranial aneurysms that traditionally have been treated through open surgical clipping [1]. In the United States, the endovascular management of intracranial aneurysms continues to increase [2, 3]. To support this trend, growing literature is demonstrating low complication rates, durable treatment, and outcomes competitive with surgical results [1, 2, 4–7].

Unlike aneurysms in other locations, the unruptured middle cerebral artery (MCA) aneurysm has several characteristics favoring surgical treatment. This includes superficial location, a familiar surgical approach, easy proximal control at the supraclinoid carotid, and minimal perforator vessels. In contrast, endovascular therapy can be somewhat more difficult in this location due to the small parent vessels, difficulty with obtaining adequate working projection views, and incorporation of branch vessels in the aneurysm. However, the endovascular management of aneurysms has evolved, and coiling of unruptured MCA aneurysms is considered an appropriate alternative to clipping for some aneurysms [8].

As the trend for endovascular management of aneurysms has grown to incorporate MCA aneurysms, recent literature has emerged to promote the surgical option as the preferred option [9, 10]. The aim of this systematic review and meta-analysis is to compare the contemporary outcome and complication rates of clipping and coiling of unruptured MCA aneurysms.

## 2. Materials and Methods

### 2.1. Search Strategy

The online database PubMed was used to perform a search of the literature between January 2004 and November 2013. The following search terms were used: (1) middle cerebral artery, (2) aneurysm, (3) coil, (4) coiling, (5) endovascular, (6) surgery, and (7) clipping. The following search string was entered into PubMed: (1) AND (2) AND ((3 OR 4 OR 5) OR (6 OR 7)).

Recently, the presented abstract by Kadkhodayan et al. [11] was also included in the endovascular group. This series, which met the rest of the inclusion criteria, was included because of its size and trial period concurrent with the other included studies.

### 2.2. Eligibility Criteria

The studies were evaluated for inclusion based on the following guidelines: (1) studies had to be consecutive case series on the treatment of MCA aneurysms using either surgical or endovascular approaches, (2) studies had to report whether treated aneurysms were unruptured or ruptured, (3) studies that examined both unruptured and ruptured aneurysms had to at least report unruptured data independent of the ruptured data, and (4) studies that examined unruptured intracranial aneurysms as a group had to report MCA aneurysm data independently. Case reports and systematic reviews were not eligible for inclusion. Studies reporting two or less MCA aneurysms were not in English and those exclusively reporting on fusiform, mycotic, or giant aneurysms were also excluded. The included studies were then further examined to ensure that outcome data were provided specifically for unruptured aneurysms, as some articles that initially discussed unruptured and ruptured aneurysms separately pooled outcome data.

### 2.3. Data Collection and Analysis

The following variables were then extracted from the included studies: (1) patient demographics; (2) unruptured and total number of aneurysms treated; (3) aneurysm locations; (4) aneurysm sizes; (5) technique and any assistive device used; (6) immediate angiographic occlusion status; (7) intraprocedural and periprocedural complications; (8) delayed angiographic occlusion status; (9) morbidity causes and rates; (10) mortality causes and rates; and (11) clinical status at follow-up. Angiographic results were classified according to Raymond's classification. We considered angiographic finding of a thrombus as a thromboembolism complication regardless of being associated or not with a clinical sequel.

Clinical outcomes were primarily reported as a modified Rankin Score (mRS). For those papers reporting outcomes as Glasgow Outcome Scores (GOS), a GOS of 4-5 was equated to an mRS of 0–2.

The R statistical software package (Vienna, Austria, V.3.0.2) was used to create a random effects model for each desired incidence rate. We took the arcsin transformed proportion as the outcome and used the inverse of the variance to weight each observation. We assumed each study came from a random sample of a larger population of similar real and hypothetical studies and used restricted maximum likelihood to estimate the variance among studies. Cochran's *Q* test [33] was used to evaluate possible heterogeneity among the rates observed in each study. The variance on the figures as the “estimated total heterogeneity” has been reported along with the *P* value for Cochran's *Q*. A *P* < 0.05 for Cochran's *Q* indicates significant variation among the study rates, suggesting some degree of noncomparability.

For each particular outcome, we compared the confidence intervals for the estimated rates for clipping and coiling where the CIs did not overlap. We concluded that the rates were significantly different at the 0.05 level and we indicated such cases in the results with “*P* < 0.05.”

## 3. Results 

### 3.1. Search Results and about Studies

The search returned 1912 articles. Of these 1912 articles, 665 were case reports and 9 were reviews, and all of them were excluded. Of the remaining 1238 articles, 101 pertained specifically to treatment of intracranial aneurysms. Of the 101 studies, 20 did not provide separate unruptured data; 4 provided only ruptured data; 19 did not provide separate MCA data; 24 provided only two or less unruptured MCA aneurysms data; 1 provided fusiform data; 10 were not in English. Thus, our search results yielded a total of 23 published series on the endovascular and surgical treatment of MCA aneurysms.

### 3.2. Studies

Including the abstract presented by Kadkhodayan et al., a total of 24 studies were included in this systematic review. Eight studies provided data on clipping of unruptured MCA aneurysms. Fifteen studies provided data on coiling of unruptured MCA aneurysms. Only one study provided data on both coiling and clipping of unruptured MCA aneurysms. Not all studies provided details on complications, angiographic outcomes, and clinical outcomes. Thus, data from the systematic review regarding these parameters are based on only a proportion of the overall number of patients included depending on the information provided in the different studies. 19 of the studies were retrospective and 5 of them were prospective (Table 1).

### 3.3. Demographics

A total of 1891 unruptured MCA aneurysms were included and, of these, 1052 aneurysms were treated by clipping and 839 aneurysms were treated by coiling. In the clipping group, the mean age was 58.2. 27.1% of patients were males and 72.9% were females. The average size of aneurysms was 5.7 mm with 95.1% of aneurysms being small (<10 mm) and 4.9% being large (10–25 mm). In the coiling group, the mean age was 53.7. Of coiled patients, 32.3% of patients were males and 67.7% were females; 84.1% of aneurysms were small and 15.9% were large (Table 1). One hundred fourteen aneurysms were treated by stent assisted coiling, and 210 were treated by balloon assisted coiling.

### 3.4. Immediate Angiographic Outcome

Angiographic results at the end of the procedure were available in 15 studies (887 aneurysms). The complete occlusion rate in the clipping group was 94.5% compared with 55.5% in the coiling group. 4.1% of the treated aneurysms in the clipping group had residual necks compared with 33% in the coiling group. Only 1.4% of aneurysms treated by clipping had residual sacs in comparison to 11.5% of those treated by coiling (*P* < 0.05). Only a small fraction (1.7%) of unruptured MCA aneurysms could not be coiled while all aneurysms were clipped in the clipping group.

### 3.5. Procedure Related Complications

Procedural complications for included studies are displayed in Table 2. The thromboembolism rate in the clipped group was 1.8% compared with 10.7% in the aneurysms treated by coiling (*P* < 0.05) (Figure 2). The parent artery occlusion rate was 0.3% in coiled group and 0.7% in the clipped group (not statistically significant). There were one TIA in the clipped group (0.2%) and 6 in the coiled group (0.9%) (not statistically significant). The intraoperative rupture rate in aneurysms treated by clipping was 1.3%, compared with 2.5% in the coiled group (not statistically significant).

### 3.6. Late Angiographic Follow-Up

**Figure 1:**
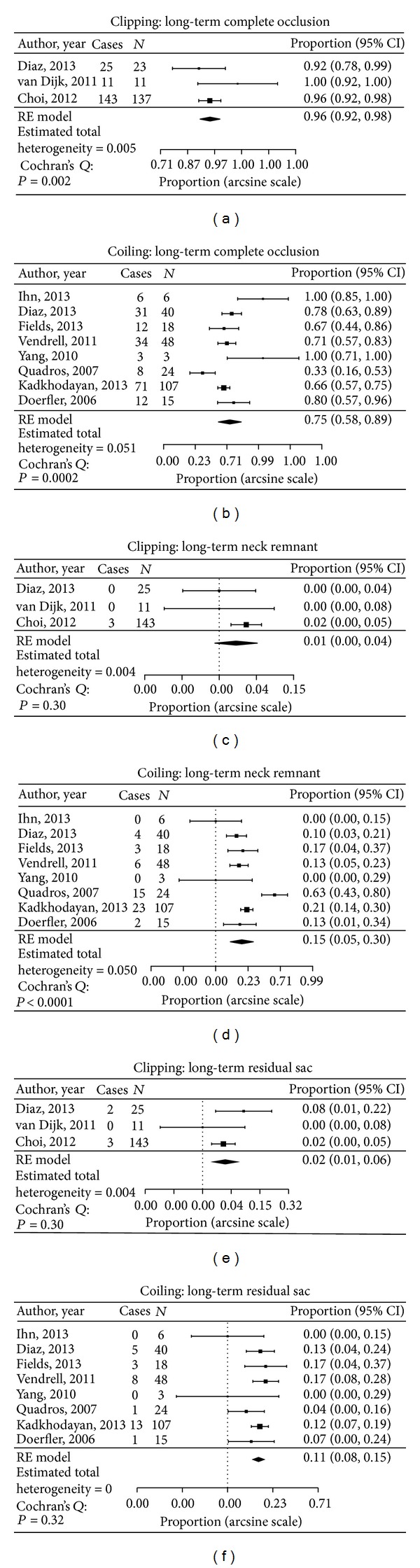
Data analysis of long-term angiographic outcome in both clipped and coiled groups.

**Figure 2:**
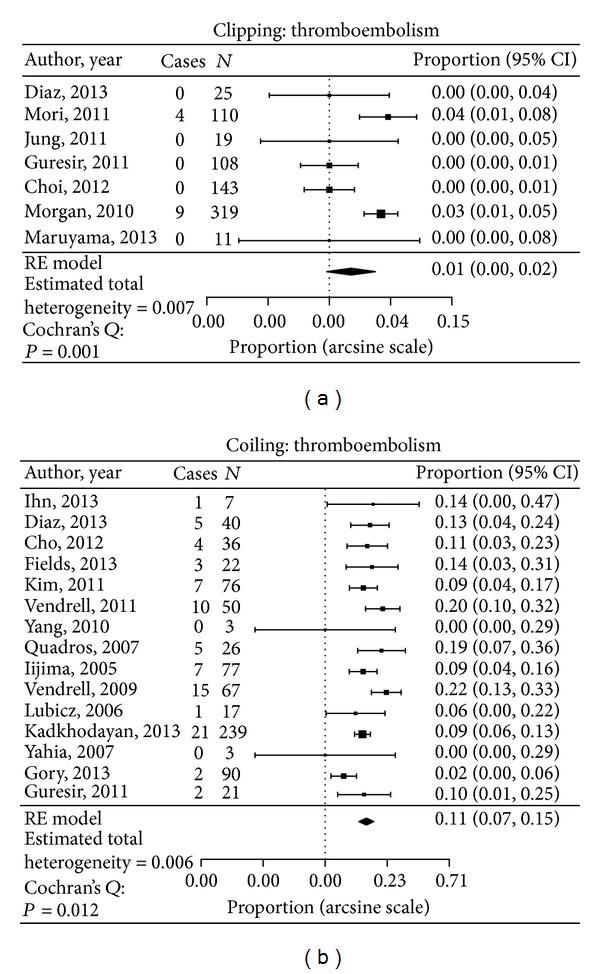
Data analysis of thromboembolism complication in both clipped and coiled groups.

Angiographic follow-up results were available in 12 studies (440 aneurysms) (Table 1). The average follow-up period in the clipped group was 6.5 months while the average follow-up period in the coiled group was 9.4 months. The complete occlusion rate in the clipping group was 95.5% compared with 67.3% in the coiled group and 1.7% of aneurysms treated by clipping had residual necks in comparison to 20.3% in the coiled group. Only 2.8% of aneurysms treated by clipping had a residual sac in comparison to 11.9% in the coiled group (*P* < 0.05) (Figure 1). No recanalization was identified with any of the aneurysms treated by clipping. In contrast, the recanalization rate for aneurysms treated by coiling was 14.3% (*P* < 0.05). In addition, 5.3% of coiled aneurysms required retreatment in contrast to only one case in the clipped group (not statistically significant).

### 3.7. Clinical Outcome

The average clinical follow-up period for the clipped group was 11 months and 9.6 months for the coiled group.

A total of 366 patients (125 clipping and 241 coiling) had outcomes reported as mRS linearly. In patients treated with clipping, 97.6% of patients had an mRS score of 0 compared to 82.9% of those treated by coiling. The percentage of patients with an mRS of 1 was 0.8% and 9.7%, respectively; with an mRS of 2 was 0.5% and 1.2%, respectively; with mRs of 3 was 0% and 2.3%, respectively; with an mRS of 4 was 0% and 2.3%, respectively; and with an mRS of 5 was 0% for both. There was no statistically significant difference between the two treatments (Table 2).

Clinical outcome was readdressed after dichotomizing mRS into good (mRS 0–2) and poor outcomes (mRS 3–5). This analysis was performed since a number of papers grouped these outcomes and this allowed a larger data inclusion (733 clipping and 461 coiling). In the clipped group, the percentage of patients with an mRS of 0–2 was 98.9% compared to 96.5% in the coiled group. There was no statistically significant difference between the two groups (Figure 4).

### 3.8. Morbidity and Mortality

**Figure 3:**
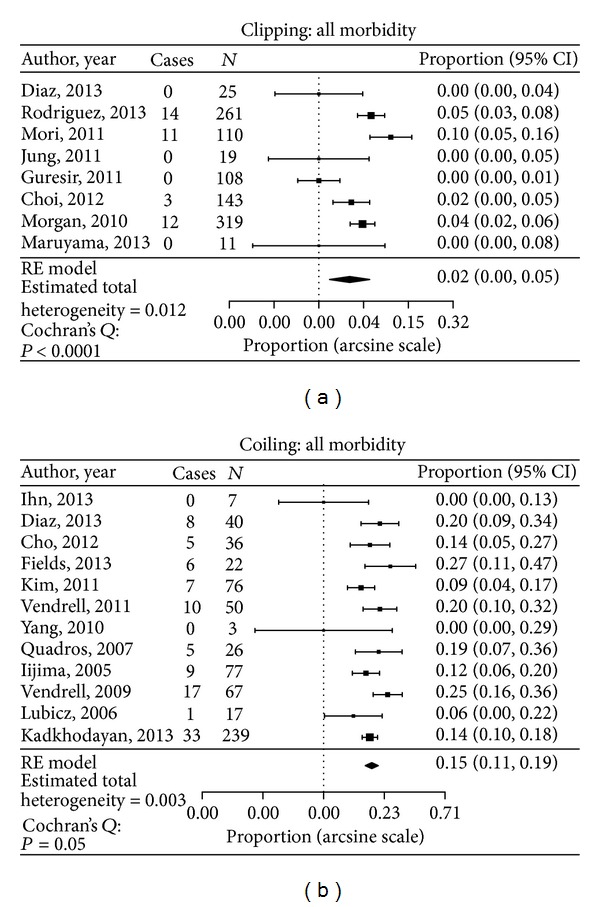
Data analysis of all morbidity cases in both clipped and coiled groups.

The overall morbidity in the clipping group was 4.6%, while, in the coiling group, it was 15.3% (*P* < 0.05) (Figure 3). Five out of 999 patients died in the clipped group (0.5%), and the mortality rate in the coiled group was 1.2% (7 deaths in 572 patients).

## 4. Discussion

The emergence of endovascular therapy has revolutionized the management of intracranial aneurysms. For aneurysms in the posterior circulation, endovascular treatment is considered preferable to open surgical clipping in most cases [34, 35].

This consensus is not generalizable to all aneurysms and the treatment risks of a particular aneurysm depend on rupture status, anatomical factors, institutional paradigm, and treating physician experience. In the case of unruptured intracranial aneurysms, the endovascular surgeon has the full armamentarium of assist devices and can easily treat even wide neck aneurysms. Despite these additional tools, the management of middle cerebral artery aneurysms is considered to be in favor of surgical treatment by many experts [9, 10, 12].

**Figure 4:**
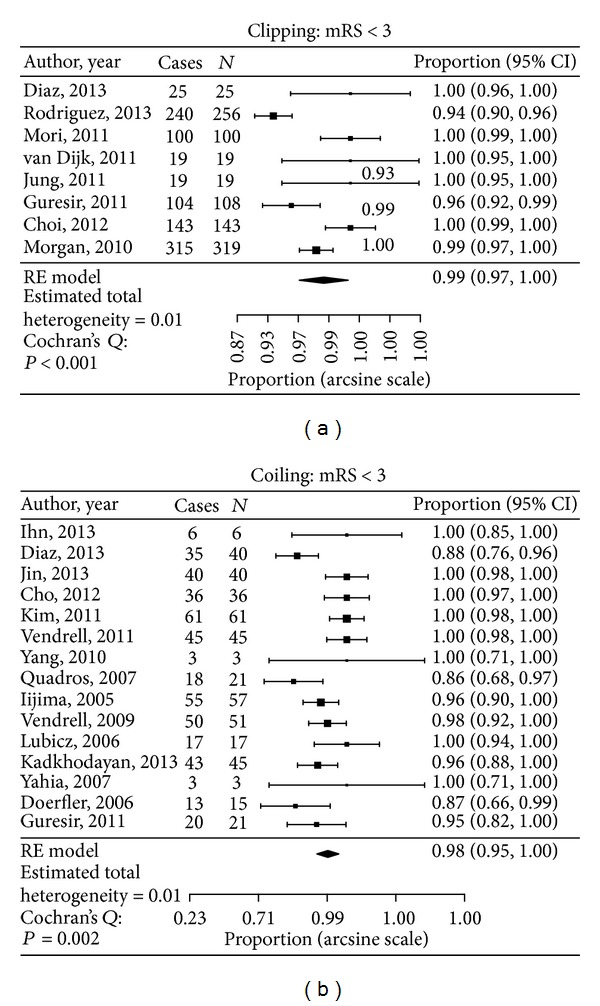
Data analysis of clinical outcome in both clipped and coiled groups.

We found that the surgical treatment of unruptured MCA aneurysms was associated with similar clinical outcomes to endovascular therapy. In the clipped group, only 2 papers reported outcomes of mRS in all categories, compared to 9 papers in the coiled group (125 clipped versus 257 coiled) [11–13, 19, 23, 25–27, 29, 30]. In many papers, outcomes were grouped into “good” or “poor” based on mRS or GOS criteria. When these results were included, the number of patients increased to 733 in the clip group and 461 in the coil group. The clinical outcomes were slightly better in clipping group when both analyses were performed but not statistically different (Figure 4). The largest difference in outcomes was in the mRS 0 and mRS 1 categories; however, since many papers only looked at mRS 0–2 as a group, the sensitivity to detect a difference in the mRS 0 to mRS 1 outcome was diluted.

When comparing clinical outcomes of coiled MCA aneurysms to aneurysms in other locations, the MCA location confers a slightly higher procedural risk. For the coiling group in our analysis, 83% (213/257, mean follow-up of 9.6 months) were an mRS 0, and an unfavorable (mRS 3–5) outcome was seen in 3.4%. In comparison, two large retrospective reviews with coiling of 916 small unruptured aneurysms, 910 (99%) patients had an mRS of 0 at the last follow-up, a better result than that of our MCA coiling group [36, 37]. This would suggest that endovascular treatment of MCA aneurysms has a higher complication rate than aneurysms in other locations.

The prospective ATENA study, reporting immediate clinical outcome of 649 patients harboring 739 unruptured intracranial aneurysms treated by endovascular coiling, indicated a change of mRS in 11 (1.7%) patients at one month and a mortality of 9 (1.4%) [38]. The ATENA study included 218 MCA aneurysms, and in subgroup analysis, the MCA location had the highest rate of thromboembolic complications compared to ACA, ICA, posterior circulation (9.6% versus 8.8% versus 4.6% versus 3.3%), and intraoperative rupture (4.1% versus 2.2% versus 1.9% versus 0.0%) [38].

In a meta-analysis of endovascular treatment of intracranial aneurysms by Naggara et al., an unfavorable outcome (mRS > 2) was seen in only 4.8% (189/5044) of patients [39]. This is very similar to our results for coiling of MCA aneurysms (3.4%, 16/461). Although unfavorable outcomes may be similar, the majority of complications in our series resulted in an mRS of 1 or 2.

A more deliberate comparison of outcomes was performed in a recently published Japanese registry of unruptured intracranial aneurysms [40]. Overall outcomes for 4573 procedures were mRS 0 in 91.7%, mRS 1 in 4.5%, and mRS 2 in 1.8%. Less than 2% of all aneurysms were mRS 3–5 at one month. Only 6.3% (301) of all aneurysms treated were MCA aneurysms. These outcomes highlight that the difference in outcomes is primarily with mRS of 0 and 1. Compared to our meta-analysis, the results for this series were better than those published for MCA aneurysms alone (mRS 0, 91.7% versus mRS 0, 83%, resp.).

Angiographic results were better reported in the coiling group than in the clipping group (261 versus 179 patients) and follow-up was for a longer period (9.4 months versus 6.5 months). With these limitations in mind, clipping offered a better angiographic result. It is not surprising that clipping yields improved angiographic results; however, incompletely coiled aneurysms likely have a very low risk of hemorrhage [41]. In the coiling group, Raymond's classes I and II were achieved in 230/261 aneurysms (88%). This result is not to be dismissed, and on top of the already low rupture risk of unruptured intracranial aneurysms, this may be adequate for protection though long-term studies are needed.

Like clinical outcomes, angiographic outcome in MCA aneurysms compared to other locations is also slightly less favorable. Im et al. [37], reporting on a series of 435 small unruptured aneurysms, were able to achieve complete or near complete occlusion in 95% of all aneurysms at treatment. Follow-up in 358/435 patients was performed at a mean of 14.2 months with 337 (94.1%) aneurysms remaining stable and 21 (5.9%) aneurysms recurring. Only 6 aneurysms were retreated. The majority of aneurysms were in the anterior circulation 390/435, and only 46/435 were MCA aneurysms.

Oishi et al. [36], reporting on 500 small unruptured intracranial aneurysms, were able to achieve complete or near complete occlusion of 79% (381/481) of aneurysms at treatment. Follow-up imaging was obtained in 427 aneurysms at an average of 31 months. Eighty-three percent (355/427) remained stable or showed progressive occlusion while recurrence occurred in 17% (72/427). Interestingly, 81 MCA aneurysms were attempted of which 9/81 (11.1%) were treatment failures, the highest failure rate of any location. The overall retreatment rate was 2.9% (14/481).

These studies seem to be in contrast to the ATENA study which showed a much lower rate of coil occlusion for all locations, complete in 63% (437/694), neck remnant in 22.5% (156/694), and aneurysm remnant 14.6% (101/694) [42]. This difference in reporting is likely accounted for by the prospective and independent review of imaging by the ATENA investigators. An independent review was performed in only three of the coiling series presented [23, 25, 26, 39].

One argument for endovascular therapy is that it is more comfortable and results in a shorter hospital stay [43–45]. This is a reasonable discerning factor when angiographic and clinical outcomes are similar and has led to the adoption of a “coil first” preference at many centers. For MCA aneurysms, obliteration is clearly better following surgical clipping than endovascular coiling. Clinical outcome between the two groups is not statistically different in this meta-analysis; however, additional publications with mRS data in the 0, 1, and 2 categories are needed for clarification. There does appear to be a trend toward higher complications with endovascular treatment of MCA aneurysms and worse clinical outcome compared to the endovascular treatment of aneurysms in other locations as well as clipping of MCA aneurysms. As a result, we favor open surgical treatment of unruptured MCA aneurysms despite the longer hospital stay and increased short-term expense. This recommendation will certainly require reassessment as more literature emerges for outcomes after endovascular treatment of MCA aneurysms.

The limitations of this review are significant. First, the patients included in the analysis were from both prospective and retrospective studies, and therefore our conclusions are subject to limitations inherent to the individual studies included. Most importantly, core lab adjudication for angiographic and clinical outcomes was not a prerequisite for inclusion in our study. Almost certainly, this results in overestimation of good angiographic results and good clinical outcomes in both groups. In addition, a portion of the included patients is from a series that has yet to be published. Lastly, when single arm studies are reviewed, a large selection bias exists. This bias likely favors the endovascular group since unfavorable aneurysms will be treated surgically.

## 5. Conclusions

Based on the results of this meta-analysis, the clinical outcomes following endovascular coiling of MCA aneurysms are not statistically different from clipping. Although there is a trend for better clinical outcome with surgery, the analysis is limited by a small amount of published data for both groups in the last 10 years. The endovascular treatment of MCA aneurysms may be associated with a higher risk than that of aneurysms in other locations.

## Figures and Tables

**Table 1:** Demographic and angiographic results of clipping and coiling studies.

Study name	Type of the study	Number of unruptured MCA aneurysms	Mean age (year)	Mean size (mm)	Average angiographic F/U, months (*N*)	Late angiographic outcome		
						R1	R2	R3
*Clipping papers *								
Diaz et al., 2012 [12]	R	30	57.7	5.8	6 (25)	23	0	2
Rodriguez-Hernandez et al., 2013 [9]	R	261	55.3	—	—	—	—	—
Mori et al., 2011 [13]	P	110	62	6.7	—	—	—	—
Van Dijk et al., 2011 [10]	P	31	52.3	—	12 (11)	11	0	0
Jung et al., 2011 [14]	R	19	72.11	6.8*	—	—	—	—
Güresir et al., 2011 [15]	P	108	51	6.5	—	—	—	—
Choi et al., 2012 [16]	R	143	57.8	4	1.5 (143)	137	3	3
Morgan et al., 2010 [17]	R	339	52.7	—	—	—	—	—
Maruyama et al., 2013 [18]	R	11	63	4.5	—	—	—	—
Total		**1052**	**58.2**	**5.7**	**6.5 (179)**	**171**	**3**	**5**
*Coiling papers *								
Ihn et al., 2013 [19]	R	7	51	4.1	7 (6)	6	0	0
Diaz et al., 2012 [12]	R	40	57.7	7.5	11.9 (40)	31	4	5
Jin, 2013 [20]	R	42	58.6	5.2*	—	—	—	—
Cho, 2012 [21]	P	36	60	—	—	—	—	—
Fields, 2013 [22]	R	22	62	—	12 (18)	12	3	3
Kim et al., 2011 [23]	R	76	59	7.8	—	—	—	—
Vendrell, 2011 [24]	R	52	52	—	14 (48)	34	6	8
Yang et al., 2010 [25]	R	3	49.7	—	5 (3)	3	0	0
Quadros et al., 2007 [26]	R	26	48	7.5	12.9 (24)	8	15	1
Iijima et al., 2005 [27]	R	77	49	7	—	—	—	—
Vendrell, 2009 [28]	R	71	49.9	6.1	—	—	—	—
Lubicz et al., 2006 [29]	P	17	51.8	—	—	—	—	—
Kadkhodayan et al., 2013 [11]	R	239	56	6.3	6 (107)	71	23	13
Yahia et al., 2008 [30]	R	3	53	10	—	—	—	—
Gory, 2013 [31]	P	90	53.2	6.4	—	—	—	—
Doerfler, 2006 [32]	R	17	47.8	6.8	6 (15)	12	2	1
Güresir et al., 2011 [15]	P	21	—	—	—	—	—	—
Total		**839**	**53.7**	**6.8**	**9.4 (261)**	**177**	**53**	**31**

Note: numbers in parentheses are *n* of aneurysms in patients who did follow-up; —: not reported. *Approximate. R: retrospective; P: prospective; R: Raymond classification for aneurysmal occlusion; R1: complete; R2: residual neck; R3: residual sac.

**Table 2:** Clinical outcome and complications of clipping and coiling studies.

Name of the study	Type of the study	Average clinical F/U, months (*N*)	Clinical outcome (mRS)								Rupture (precedual)	Mortality	Morbidity	Thromboembolism	Parent artery occlusion	Recanalization	Retreatment
			0	1	2	3	4	5	0–2	3–5							
*Clipping papers *																	
Diaz et al., 2012 [12]	R	6 (25)	23	0	2	0	0	0	25	0	0	0	0	0	0	0	0
Rodriguez-Hernandez et al., 2013 [9]	R	—	—	—	—	—	—	—	—	—	9	5	14	—	—	—	0
Mori et al., 2011 [13]	P	3 (100)	99	1	0	0	0	0	100	0	0	0	11	4	—	—	1
van Dijk et al., 2011 [10]	P	56.4 (19)	—	—	—	—	—	—	19	0	0	0	—	—	—	—	—
Jung, 2011 [14]	R	3 (19)	—	—	—	—	—	—	19	0	0	0	0	0	0	0	0
Güresir et al., 2011 [15]	P	6 (108)	—	—	—	—	—	—	104	4	0	0	0	0	0	—	0
Choi, 2012 [16]	R	1.5 (143)	—	—	—	—	—	—	143	0	0	0	3	0	0	—	—
Morgan, 2010 [17]	R	1.5 (319)	—	—	—	—	—	—	315	4	—	0	12	9	2	—	—
Maruyama, 2013 [18]	R	—	—	—	—	—	—	—	—	—	0	0	0	0	0	0	0
Total		**11**	**122**	**1**	**2**	**0**	**0**	**0**	**725**	**8**	**9**	**5**	**40**	**13**	**2**	**0**	**1**
*Coiling papers *																	
Ihn et al., 2013 [19]	R	7 (6)	6	0	0	0	0	0	6	0	0	0	0	1	—	0	0
Diaz et al., 2012 [12]	R	6 (40)	22	13	0	2	3	0	35	5	1	0	8	5	1	6	
Jin, 2013 [20]	R	29.5 (40)	—	—	—	—	—	—	40*	0	—	0	—	—	—	—	—
Cho, 2012 [21]	P	0.25 (36)	—	—	—	—	—	—	36*	0	1	0	5	4	0	4	0
Fields, 2013 [22]	R	—	—	—	—	—	—	—	—	—	1	0	6	3	1	2	1
Kim et al., 2011 [23]	R	25 (63)	61	0	0	0	0	0	61	0	1	2	7	7	0	9	3
Vendrell, 2011 [24]	R	6 (45)	—	—	—	—	—	—	45*	0	0	0	10	10	0	7	5
Yang et al., 2010 [25]	R	20 (3)	2	1	0	0	0	0	3	0	0	0	0	0	0	0	0
Quadros et al., 2007 [26]	R	12.9 (22)	14	2	2	2	1	0	18	3	0	1	5	5	0		1
Iijima et al., 2005 [27]	R	3 (58)	55	0	0	1	1	0	55	2	1	1	9	7	1	6	—
Vendrell, 2009 [28]	R	12 (51)	—	—	—	—	—	—	50*	1	2	0	17	15	0	13	4
Lubicz et al., 2006 [29]	P	1 (17)	16	1	0	0	0	0	17	0	—	0	1	1	0	—	0
Kadkhodayan et al., 2013 [11]	R	6 (45)	34	8	1	1	1	0	43	2	4	0	33	21	—	—	—
Yahia et al., 2008 [30]	R	3 (3)	3*	0	0	0	0	0	3	0	0		0	0	—	—	—
Gory, 2013 [31]	P	—	—	—	—	—	—	—	—	—	8	2	—	2	—	—	—
Doerfler, 2006 [32]	R	6 (16)	—	—	—	—	—	—	13*	2	0	1	—	—	—	1	1
Güresir et al., 2011 [15]	P	6 (21)	—	—	—	—	—	—	20	1	—	0	—	2	—	—	—
Total			**213**	**25**	**3**	**6**	**6**	**0**	**445**	**16**	**19**	**7**	**101**	**83**	**3**	**48**	**15**

Note: numbers in parentheses are *n* of aneurysms in patients who did follow-up; —: not reported, P: prospective, and R: retrospective; *mRS estimated from GOS.
